# ETAS®50 Attenuates Ultraviolet-B-Induced Interleukin-6 Expression by Suppressing Akt Phosphorylation in Normal Human Dermal Fibroblasts

**DOI:** 10.1155/2018/1547120

**Published:** 2018-07-05

**Authors:** Ken Shirato, Tomoko Koda, Jun Takanari, Junetsu Ogasawara, Takuya Sakurai, Hideki Ohno, Takako Kizaki

**Affiliations:** ^1^Department of Molecular Predictive Medicine and Sport Science, Kyorin University School of Medicine, 6-20-2 Shinkawa, Mitaka, Tokyo 181-8611, Japan; ^2^Faculty of Nursing, Tokyo Healthcare University, 2-5-1 Higashigaoka, Meguro, Tokyo 152-8558, Japan; ^3^Amino Up Chemical Co. Ltd., 363-32 Shin-ei, Kiyota, Sapporo, Hokkaido 004-0839, Japan; ^4^Department of Health Science, Asahikawa Medical University, 2-1-1-1 Midorigaoka-Higashi, Asahikawa, Hokkaido 078-8510, Japan; ^5^Social Medical Corporation, The Yamatokai Foundation, 1-13-12 Nangai, Higashiyamato, Tokyo 207-0014, Japan

## Abstract

We recently reported that ETAS 50, a standardized extract from the* Asparagus officinalis* stem, exerted anti-inflammatory effects on ultraviolet-B- (UV-B-) irradiated normal human dermal fibroblasts (NHDFs) by inhibiting nuclear factor-*κ*B p65 nuclear import and the resulting interleukin-1*β* (IL-1*β*) expression. To further elucidate the antiphotoaging potency of ETAS 50, we examined the anti-inflammatory effects on UV-B-irradiated NHDFs by focusing on the stress-activated mitogen-activated protein kinase (MAPK) and Akt signaling pathways. NHDFs were treated with 1 mg/mL of ETAS 50 or dextrin (vehicle control) after UV-B irradiation (20 mJ/cm^2^) for different time periods. Phosphorylation levels of c-Jun N-terminal kinase (JNK), p38 MAPK, and Akt were analyzed by western blotting. IL-6 mRNA levels were analyzed by real-time polymerase chain reaction. UV-B-irradiated NHDFs showed increased phosphorylation levels of JNK, p38 MAPK, and Akt, as well as increased mRNA levels of IL-6. ETAS 50 treatment after UV-B irradiation suppressed the increased phosphorylation levels of Akt without affecting those of JNK and p38 MAPK. ETAS 50 as well as Akt inhibitor Perifosine repressed UV-B irradiation-induced IL-6 mRNA expression. These results suggest that ETAS 50 treatment represses UV-B irradiation-induced IL-6 expression by suppressing Akt phosphorylation. The present findings demonstrate the potential of ETAS 50 to prevent photoaging by attenuating UV-B irradiation-induced proinflammatory responses in skin fibroblasts.

## 1. Introduction

Premature skin aging (photoaging), characterized by deep wrinkle formation, is accelerated by inflammatory responses in ultraviolet- (UV-) irradiated skin cells [1]. UV rays that reach the surface of earth consist of short-wavelength UV-B (280–315 nm; 5%) and long-wavelength UV-A (315–400 nm; 95%). Both types of UV rays activate a variety of intracellular signaling pathways, which mediate production of proinflammatory mediators in skin cells, including keratinocytes and fibroblasts. These cellular events are involved in accelerating extracellular matrix breakdown and causing deep wrinkle formation by inducing leukocyte migration, activation, and reactive oxygen species (ROS) production in skin tissues. Among the signaling pathways, nuclear factor-*κ*B (NF-*κ*B) signaling mediates UV-B irradiation-induced production of proinflammatory cytokines, inducible nitric oxide synthase (iNOS), matrix metalloproteinase-1 (MMP-1), and MMP-3 in primary human dermal fibroblasts [2–4].

UV irradiation also activates the stress-activated mitogen-activated protein kinase (MAPK) signaling proteins, such as c-Jun N-terminal kinase (JNK) and p38 MAPK [5–7]. These kinases regulate the transactivation of proinflammatory cytokines in murine skin tissues in a manner similar to NF-*κ*B [5–7]. In addition to the MAPK signaling proteins, phosphorylation of protein kinase B/Akt is induced when human skin keratinocytes and fibroblasts are irradiated with UV rays [8, 9]. There are a limited number of studies that demonstrate that Akt mediates UV irradiation-induced production of proinflammatory cytokines in skin cells. However, it is reported that several chemical factors, such as phosphatidic acid, Shiga toxin 1, and a toll-like receptor 5 ligand flagellin, induce proinflammatory cytokine production via Akt signaling in macrophages [10–12]. Since proinflammatory mediators produced by UV-irradiated skin cells promote infiltration and ROS production by immune cells in the skin and the resulting collagen breakdown causes deep wrinkle formation, suppressing the proinflammatory signaling in UV-irradiated skin cells is crucial in preventing photoaging and maintaining skin health.

ETAS 50 is a standardized extract from the* Asparagus officinalis* stem, produced by Amino Up Chemical Co. Ltd. (Sapporo, Japan). It is a unique eco-friendly functional food that attenuates sleep deprivation-induced stress responses, promotes good sleep, and increases salivary secretory immunoglobulin A levels in mice and humans [13–15]. In addition to its antistress effects, later studies demonstrated the beneficial effects of ETAS 50 on brain functions, including the ability to ameliorate cognitive impairment in senescence-accelerated mice [16], protect neuronal PC-12 cells from amyloid *β*-induced cytotoxicity [17], and improve learning ability in young healthy rats [18].

We recently reported that ETAS 50 suppresses hydrogen peroxide-induced interleukin-12*α* (IL-12*α*) and iNOS mRNA expression in murine skin L929 fibroblasts and UV-B irradiation-induced IL-1*β* mRNA expression in normal human dermal fibroblasts (NHDFs), by inhibiting nuclear import of p65 [19, 20]. Moreover, ETAS 50 attenuated hydrogen peroxide-induced MMP-9 expression by suppressing the phosphorylation of JNK and its downstream transcription factor c-Jun [21]. Previous findings suggest that ETAS 50 exhibits antiphotoaging effects by suppressing proinflammatory signaling in irradiated skin cells. To further elucidate the antiphotoaging potency, this study aimed to clarify the anti-inflammatory effects of ETAS 50 on UV-B-irradiated NHDFs, focusing on the stress-activated MAPK and Akt signaling pathways.

## 2. Materials and Methods

### 2.1. Preparation of ETAS 50

In the present study, the same batch of ETAS 50 (Amino Up Chemical Co. Ltd.) was used as in previous studies [19–21]. The procedure for preparing ETAS 50 was described previously [13, 14]. Briefly, fresh bottom parts of the* Asparagus officinalis* stem were boiled in water at 121°C under 0.12 MPa for 45 min. The boiled stem and resulting extract were treated with cellulase and pectinase, both of which are widely used in the food industry. After these enzymes were inactivated by incubation at 121°C for 20 min, the extract was centrifuged, and the resulting supernatant was mixed with dextrin (Pinedex; Matsutani Chemical Industry, Hyogo, Japan) as a filler. The mixture was then concentrated* in vacuo* at 105°C, sterilized at 121°C for 45 min, and finally spray-dried to produce an ETAS 50 powder consisting of 52.6% ETAS 50 and 47.4% dextrin. Component analysis revealed that the ETAS 50 powder was composed of 86.5% carbohydrates, 7.1% proteins, 2.9% ash, 1.0% lipids, and 2.5% moisture. In this study, the concentrations of ETAS 50 excluding dextrin are indicated.

### 2.2. Cell Culture and UV-B Irradiation

Cell culture and UV-B irradiation were conducted as described previously [19]. NHDFs from an adult donor (age: 47; sex: female; race: Caucasian; PromoCell, Heidelberg, Germany) were cultured in Fibroblast Growth Medium 2 (PromoCell) supplemented with 2% fetal calf serum, 1 ng/mL of basic fibroblast growth factor, and 5 *μ*g/mL of insulin. The cultures were maintained at 37°C in a humidified incubator containing 5% CO_2_ in air. All experiments were conducted using cells at passage 4. Prior to the initiation of experiments, NHDFs were seeded into 6-well plates at a density of 1.5 × 10^4^ cells/cm^2^ and cultured for 24 h. UV-B irradiation was administered with a UVM-57 Handheld UV Lamp (6 watts, 302 nm; UVP, LLC, Upland, CA, USA), which was applied at distance of 7.5 cm from the cells. Irradiance was measured with a UV light meter UV-340C (CUSTOM, Tokyo, Japan). To test the effect of UV-B alone, the cells were washed twice with phosphate-buffered saline (PBS) and then irradiated under a thin layer of PBS with UV-B for 30 s to obtain a total dose of 20 mJ/cm^2^. Immediately after irradiation, the cells were cultured in complete medium for different time periods. ETAS 50 or dextrin vehicle control was directly dissolved in complete medium to produce a final concentration of 1 mg/mL. Immediately after UV-B irradiation, the cells were cultured in supplemented medium for different time periods. Treatment with the NF-*κ*B inhibitor JSH-23 (Sigma-Aldrich, St. Louis, MO, USA) or Akt inhibitor Perifosine (Cell Signaling Technology, Danvers, MA, USA) was also performed immediately after UV-B irradiation.

### 2.3. Western Blotting

Total protein was extracted from cells using RIPA Buffer (Sigma-Aldrich) supplemented with a Complete Protease Inhibitor Cocktail Tablet (Roche Life Science, Basel, Switzerland) and Phosphatase Inhibitor Cocktail (Nacalai Tesque, Kyoto, Japan). Protein concentrations were determined using a BCA Protein Assay Kit (Pierce Biotechnology, Rockford, IL, USA). Protein samples (10 *μ*g) were denatured by incubation at 95°C for 5 min in sample buffer containing 75 mM Tris-HCl (pH 6.8), 0.6% sodium dodecyl sulfate, 15% glycerol, 7.5%  *β*-mercaptoethanol, and 9 *μ*g/ml of bromophenol blue. The denatured proteins were separated by electrophoresis using a sodium dodecyl sulfate-polyacrylamide gel and then transferred to a polyvinylidene difluoride membrane (GE Healthcare, Little Chalfont, UK). Each membrane was blocked with 5% bovine serum albumin, and primary antibodies against phospho-JNK (Thr183/Tyr185), JNK, phospho-p38 MAPK (Thr180/Tyr182), p38 MAPK, phospho-Akt (Ser473), or Akt (Cell Signaling Technology) were applied to the membrane at a 1 : 1,000 dilution overnight. After washing, the membranes were incubated with secondary antibody (horseradish peroxidase-conjugated AffiniPure Mouse Anti-Rabbit IgG; Jackson ImmunoResearch Laboratories, West Grove, PA, USA) at a 1 : 20,000 dilution for 30 min. After washing, the membranes were finally incubated with Clarity Western ECL Substrate (Bio-Rad Laboratories, Hercules, CA, USA) and exposed to X-ray film. The density of each protein band was quantified using ImageJ software (National Institutes of Health, Bethesda, MD, USA). Phosphorylation levels of JNK, p38 MAPK, and Akt were calculated as the ratios of their phosphorylated forms to total amounts.

### 2.4. Reverse Transcription and Real-Time Polymerase Chain Reaction (PCR)

Total RNA was extracted from cells using RNAiso Plus reagent (TaKaRa Bio, Shiga, Japan). One microgram of total cellular RNA was converted to cDNA using the PrimeScript 1st-Strand cDNA Synthesis Kit (Takara Bio). cDNA (1 *μ*L) was amplified with the FastStart Universal Probe Master (Roche Life Science) using a 7500 Real-Time PCR System (Thermo Fisher Scientific, Waltham, MA, USA). The PCR conditions were 50°C for 2 min and 95°C for 15 s, followed by 45 cycles at 95°C for 15 s and 60°C for 1 min. The primer sequences and fluorescent probes were as follows: IL-6, forward 5′-GCC CAG CTA TGA ACT CCT TCT-3′, reverse 5′-CTT CTC CTG GGG GTA CTG G-3′, Universal Probe #68 (Roche Life Science); 18S, forward 5′-AAA TCA GTT ATG GTT CCT TTG GTC-3′, reverse 5′-GCT CTA GAA TTA CCA CAG TTA TCC AA-3′, Universal Probe #55 (Roche Life Science). The expression levels of IL-6 mRNA were calculated as the ratio of its value to that of 18S rRNA as an internal control.

### 2.5. Statistical Analysis

The experimental data are presented as mean ± standard errors of the mean (SEM). Differences between two groups were assessed using Student's* t*-test. Comparisons among at least three groups were tested by one-way analysis of variance (ANOVA), and then post hoc comparisons to determine significant differences between groups were performed using Tukey's test. Differences were considered statistically significant when *p* values were less than 0.05.

## 3. Results and Discussion

**Figure 1:**
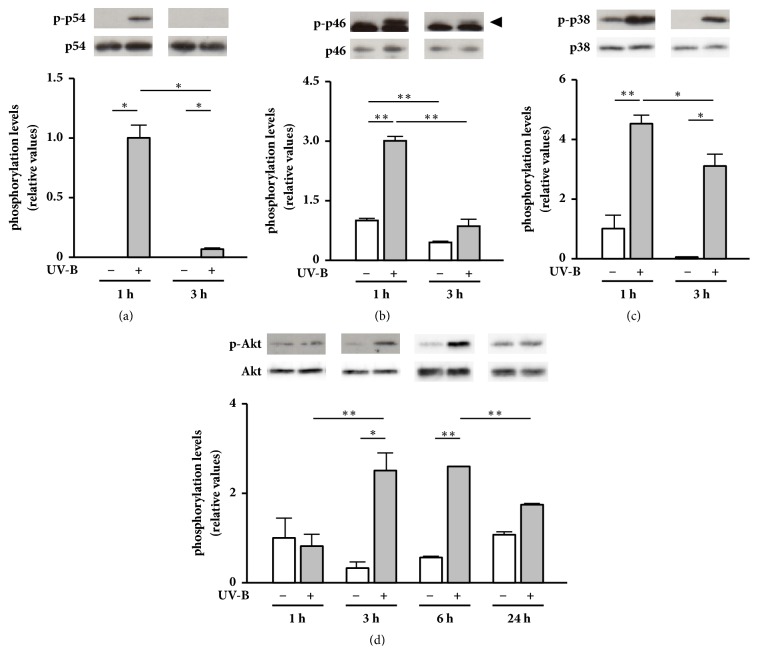
Time course changes in phosphorylation levels of stress-activated MAPKs and Akt after UV-B irradiation in NHDFs. Cells were cultured for 3–24 h after UV-B irradiation (20 mJ/cm^2^). (Upper panels) phosphorylated and total amounts of JNK subunits p54 (a) and p46 (b), p38 MAPK (c), and Akt (d) were detected by western blotting. (b) An arrow indicates p-p46. Nonspecific, strong luminescence under p-p46 is derived from an unknown protein. (Lower panels) phosphorylation levels were calculated as the ratios of the phosphorylated forms to total amount, mean ± SEM (*n* = 3). ^*∗*^*p* < 0.05 and ^*∗∗*^*p* < 0.01 (by one-way ANOVA and Tukey's test).

We first analyzed the time course changes in phosphorylation levels after UV-B irradiation in NHDFs. Phosphorylation levels of JNK subunits p54 and p46 transiently increased 1 h after UV-B irradiation and subsequently returned to nonirradiated levels after 3 h of culture (Figures 1(a) and 1(b)). Phosphorylation levels of p38 MAPK increased 1 h after UV-B irradiation and significantly decreased after 3 h of culture (Figure 1(c)). Although phosphorylation levels of Akt were not changed 1 h after UV-B irradiation, the levels significantly increased during 3–6 h of culture and returned to nonirradiated levels after 24 h of culture (Figure 1(d)). These results suggest that stress-activated MAPKs, including JNK and p38 MAPK, are rapidly phosphorylated within 1 h following UV-B irradiation, whereas Akt phosphorylation levels gradually increased after 3 h of culture. Several studies have shown that UV-B irradiation induces the phosphorylation of JNK, p38 MAPK, and Akt in skin cells and tissues [5–9]. Therefore, phosphorylation of these signaling proteins in NHDFs in the present study showed a similar tendency to that in skin cells and tissues irradiated with UV rays in the previous studies [5–9].

**Figure 2:**
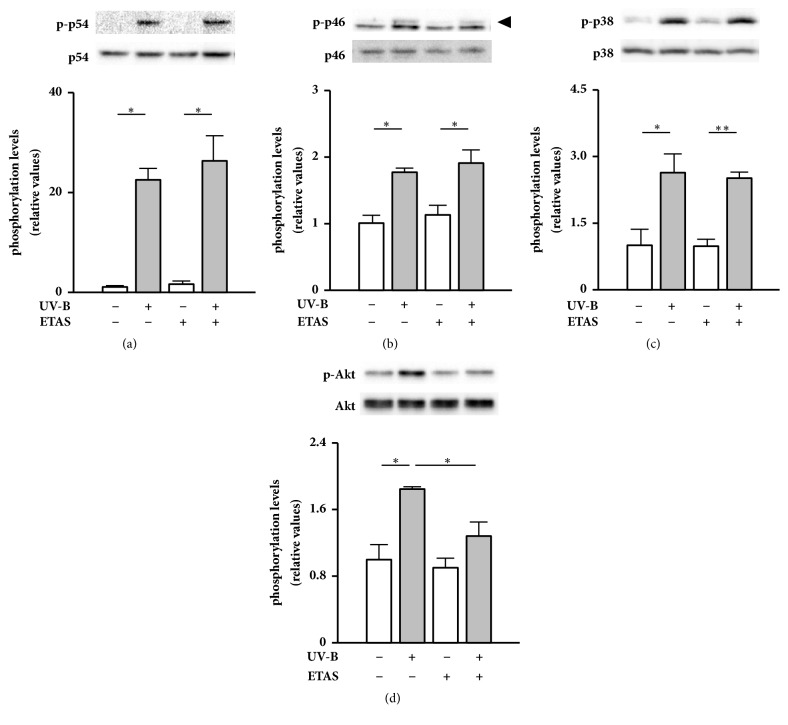
Effects of ETAS 50 treatment on UV-B irradiation-induced phosphorylation of stress-activated MAPKs and Akt in NHDFs. Cells were treated with 1 mg/mL of ETAS 50 or dextrin (vehicle control) for 1 or 3 h after UV-B irradiation (20 mJ/cm^2^). After 1 h of culture, phosphorylated and total amounts of JNK subunits p54 (a) and p46 (b), as well as p38 MAPK (c), were detected by western blotting; those of Akt (d) were analyzed after 3 h of culture. (b) An arrow indicates p-p46. Nonspecific, strong luminescence under p-p46 is derived from an unknown protein. Phosphorylation levels were calculated as the ratios of the phosphorylated forms to total amount, mean ± SEM (*n* = 4). ^*∗*^*p* < 0.05 and ^*∗∗*^*p* < 0.01 (by one-way ANOVA and Tukey's test).

We next examined whether ETAS 50 suppresses UV-B irradiation-induced phosphorylation of stress-activated MAPKs in NHDFs. ETAS 50 treatment after UV-B irradiation did not influence phosphorylation levels of both JNK subunits and p38 MAPK after 1 h of culture (Figures 2(a)–2(c)). However, we recently reported that simultaneous ETAS 50 treatment attenuates hydrogen peroxide-induced phosphorylation of JNK and the downstream transcription factor c-Jun after 1 h of culture in murine L929 skin fibroblasts [21]. Although it is not clear why ETAS 50 did not influence UV-B-induced JNK phosphorylation (Figures 2(a) and 2(b)) but attenuated hydrogen peroxide-induced JNK phosphorylation [21], it is possible that UV-B irradiation induced JNK phosphorylation more rapidly than hydrogen peroxide treatment did. It is also possible that a time lag between UV-B irradiation and ETAS 50 treatment eliminated the suppressive effects on JNK phosphorylation. Therefore, ETAS 50 pretreatment may be necessary to suppress UV-B irradiation-induced phosphorylation of stress-activated MAPKs.

On the other hand, ETAS 50 treatment significantly suppressed UV-B irradiation-induced increases in Akt phosphorylation levels after 3 h of culture, without affecting basal phosphorylation levels (Figure 2(d)). These results suggest that ETAS 50 inhibits UV-B irradiation-responsive Akt phosphorylation in NHDFs. Unlike stress-activated MAPKs, phosphorylation levels of Akt gradually increased after UV-B irradiation (Figures 1(a)–1(d)), which might enable ETAS 50 ingredients to sufficiently penetrate and act on cells, resulting in suppressed Akt phosphorylation. Akt signaling is a master regulator for cellular proliferation, survival, and energy metabolism [22]. A previous study demonstrated that oral acute and subacute ETAS 50 administration has no significant side effects on food consumption, body weight, mortality, urinalysis, hematology, biochemistry, necropsy, organ weight, and histopathology in rats [23]. The lack of side effects may be due to ETAS 50 having minimal influence on basal Akt phosphorylation.

In this study, western blot analysis could not detect phosphorylation of phosphoinositide 3-kinase (PI3-K) subunits p85 and p55, 1 and 3 h after UV-B irradiation in NHDFs (data not shown). This suggests that UV-B-induced Akt phosphorylation in the NHDFs is not mediated by activated PI3-K. By contrast, it has been reported that murine skin irradiated with UV-B (180 mJ/cm^2^) showed increased Akt phosphorylation, which was accompanied by increased PI3-K activity, which directly catalyzes Akt phosphorylation [24]. It is conceivable that the mechanisms underlying UV-B irradiation-induced Akt phosphorylation are different between* in vitro* and* in vivo* levels. Indeed, in addition to PI3-K, a diverse group of tyrosine and serine/threonine kinases that directly activate Akt were identified [25]. Furthermore, the physical association between 3-phosphoinositide-dependent kinase 1 and its acceptor substrate Akt is enough to phosphorylate Akt, even if PI3-K is not activated and Akt is not localized at the plasma membrane [26].

Our previous study showed that ETAS 50 could not reverse hydrogen peroxide-induced increase in protein carbonylation levels, an index of protein oxidation, which suggested that the suppressive effects on hydrogen peroxide-induced JNK and c-Jun phosphorylation are not mediated by its capability to scavenge ROS [21]. Therefore, ETAS 50 is likely to contain active compounds to directly modulate the activities of kinases other than PI3-K or phosphatases that regulate Akt phosphorylation and dephosphorylation. It will be necessary to identify the active compounds in ETAS 50 and analyze their biological activities to further understand the underlying mechanism of its suppressive effects on UV-B irradiation-responsive Akt phosphorylation.

**Figure 3:**
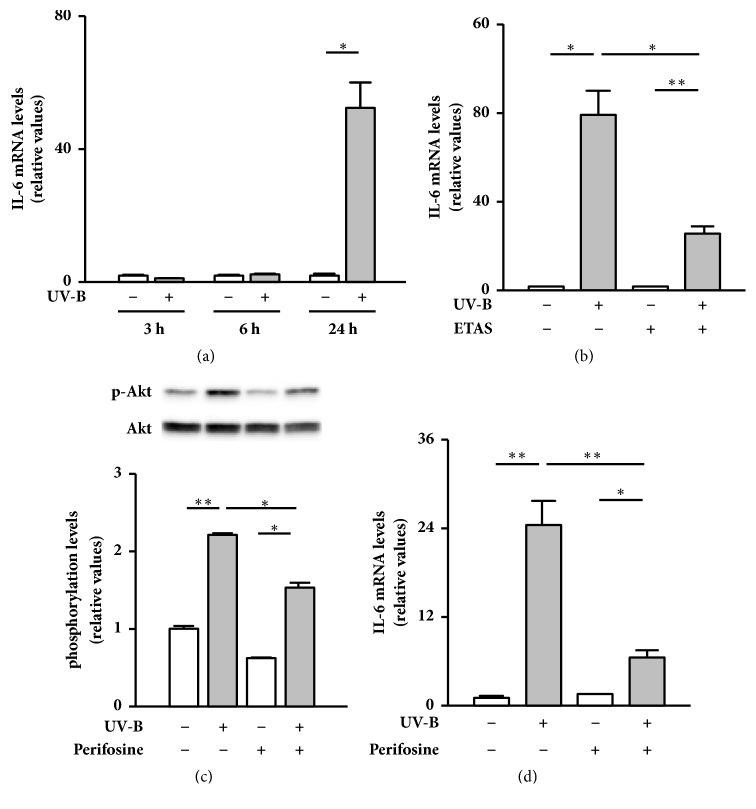
Effect of ETAS 50 treatment on UV-B irradiation-induced IL-6 mRNA expression in NHDFs. (a) Time course changes in IL-6 mRNA levels after UV-B irradiation. Cells were cultured for 3–24 h after UV-B irradiation (20 mJ/cm^2^). The mRNA levels of IL-6 were analyzed by real-time PCR. The expression levels of IL-6 mRNA were calculated as the ratio of its value to that of 18S rRNA, mean ± SEM (*n* = 3). ^*∗*^*p* < 0.05 (by Student's* t*-test). (b) Effects of ETAS 50 on UV-B irradiation-induced IL-6 mRNA expression. Cells were treated with 1 mg/mL of ETAS 50 or dextrin (vehicle control) for 24 h after UV-B irradiation (20 mJ/cm^2^). The mRNA levels of IL-6 were analyzed as described above. (c) Effects of Perifosine on the UV-B irradiation-induced Akt phosphorylation. The cells were treated with 20 *μ*M Perifosine or H_2_O (vehicle control) for 3 h after UV-B irradiation (20 mJ/cm^2^). Phosphorylated and total amounts of Akt were detected by western blotting. Phosphorylation levels were calculated as the ratios of the phosphorylated forms to total amount. (d) Effects of Perifosine on the UV-B irradiation-induced IL-6 mRNA expression. The cells were treated with 20 *μ*M Perifosine or H_2_O (vehicle control) for 24 h after UV-B irradiation (20 mJ/cm^2^). The mRNA levels of IL-6 were analyzed as described above, mean ± SEM (*n* = 3). ^*∗*^*p* < 0.05 and ^*∗∗*^*p* < 0.01 (by one-way ANOVA and Tukey's test).

Finally, to investigate anti-inflammatory properties of ETAS 50, we analyzed the time course effects of UV-B irradiation on the expression levels of IL-6 mRNA in NHDFs. Although mRNA levels of IL-6 were similar between cells irradiated with and without UV-B rays after 3 h and 6 h of culture, UV-B-irradiated cells had dramatically higher amounts of IL-6 mRNA than nonirradiated cells after 24 h of culture (Figure 3(a)). When the cells were treated with ETAS 50 after UV-B irradiation, the increase in IL-6 mRNA levels was markedly suppressed (Figure 3(b)). Moreover, treatment with an NF-*κ*B nuclear translocation inhibitor JSH-23 showed a propensity to attenuate the UV-B irradiation-induced IL-6 mRNA expression, but the effect was not significant (Supplementary Figure 1). This finding suggests that NF-*κ*B plays only a minor role in inducing IL-6 transactivation. On the other hand, the UV-B irradiation-induced Akt phosphorylation, concomitant with IL-6 mRNA expression, was markedly suppressed by subsequent treatment with Perifosine (Figures 3(c) and 3(d)). The agent is an inhibitor of Akt that does not directly affect PI3-K [27]. This inhibitory assay indicates that Akt plays a crucial role in UV-B-induced IL-6 transactivation. These results demonstrate that ETAS 50 suppresses UV-B irradiation-induced IL-6 mRNA expression by inhibiting Akt phosphorylation.

There are several reports that UV rays activate skin cells to produce a variety of proinflammatory cytokines, such as IL-6 and IL-1*β* [28–30]. UV rays-induced proinflammatory responses in skin cells, including dermal fibroblasts, contribute to accelerating premature skin aging. Thus, suppressing the proinflammatory responses in UV-irradiated skin cells is crucial in preventing photoaging and maintaining skin health. In addition to our present finding, we also recently reported that ETAS 50 markedly suppressed UV-B irradiation-induced IL-1*β* transactivation in NHDFs by inhibiting nuclear translocation of the NF-*κ*B p65 subunit [19]. Moreover, a recent study has shown that ETAS 50 attenuated IL-1*β*-induced iNOS expression in rat primary hepatocytes [31]. Therefore, ETAS 50 has the potential to prevent proinflammatory responses induced directly by UV-B irradiation and indirectly by cytokines such as IL-1*β* and IL-6 that are produced by UV-B-irradiated skin fibroblasts.

Ongoing analysis suggested that ETAS 50 contains proline-containing 3-alkyldiketopiperazine derivatives as its active compounds. When ETAS 50 was orally administered, it attenuated sleep deprivation-induced stress responses, promotes good sleep, and increases salivary secretory immunoglobulin A levels in mice and humans [13–15]. These previous findings suggest a possibility that the compounds or their metabolites are delivered to central nervous system via circulation. However, it may have a limitation, whether the active compounds are really delivered to peripheral skin tissues when ETAS 50 is orally taken as functional food. Although ETAS 50 certainly showed unique anti-inflammatory actions on UV-B-irradiated NHDFs in* in vitro* studies, further study is necessary to clarify whether its direct application as lotion or ointment is useful for maintaining skin health.

## 4. Conclusion

Our previous study showed that ETAS 50 attenuated UV-B-irradiation-induced IL-1*β* expression by inhibiting NF-*κ*B nuclear import in NHDFs [19]. The present study demonstrated ETAS 50 to attenuate UV-B-induced IL-6 expression by suppressing Akt phosphorylation. These findings suggest that ETAS 50 has potential for preventing skin aging through anti-inflammatory effects. Therefore, this readily available, inexpensive, and eco-friendly functional food may be a useful component in dermatologic prophylactic strategies for maintaining skin health and function.

## Data Availability

The experimental data used to support the findings of this study are included within the article and supplementary information file.

## Supplementary Materials

Supplementary Materials Supplementary Figure 1: effect of an NF-*κ*B inhibitor JSH-23 treatment on UV-B irradiation-induced IL-6 mRNA expression in NHDFs. Cells were treated with 10 *μ*M JSH-23 or dimethylsulfoxide vehicle control for 24 h after UV-B irradiation (20 mJ/cm^2^). The mRNA levels of IL-6 were analyzed by real-time PCR. The expression levels of IL-6 mRNA were calculated as the ratio of its value to that of 18S rRNA, mean ± SEM (*n* = 3). ^*∗*^*p* < 0.05 and ^*∗∗*^*p* < 0.01 (by one-way ANOVA and Tukey's test).
