# Supplementary material for: ETAS®50 Attenuates Ultraviolet-B-Induced Interleukin-6 Expression by Suppressing Akt Phosphorylation in Normal Human Dermal Fibroblasts
Source: Evid Based Complement Alternat Med. 2018 Jul 5;2018:1547120. doi: 10.1155/2018/1547120 (PMC6077605; doi:10.1155/2018/1547120)
Supplement: Supplementary Materials — Supplementary Figure 1: effect of an NF-κB inhibitor JSH-23 treatment on UV-B irradiation-induced IL-6 mRNA expression in NHDFs. Cells were treated with 10 μM JSH-23 or dimethylsulfoxide vehicle control for 24 h after UV-B irradiation (20 mJ/cm2). The mRNA levels of IL-6 were analyzed by real-time PCR. The expression levels of IL-6 mRNA were calculated as the ratio of its value to that of 18S rRNA, mean ± SEM (n = 3). ∗p < 0.05 and ∗∗p < 0.01 (by one-way ANOVA and Tukey's test). [file 1547120.f1.pdf]

Evidence-Based Complementary and Alternative Medicine

Research Article

ETAS®50 Attenuates Ultraviolet-B-Induced Interleukin-6 Expression by Suppressing Akt Phosphorylation in Normal Human Dermal Fibroblasts

Ken Shirato,[1] Tomoko Koda,[2] Jun Takanari,[3] Junetsu Ogasawara,[4] Takuya Sakurai,[1] Hideki Ohno,[5] and Takako Kizaki[1]

[1]*Department of Molecular Predictive Medicine and Sport Science, Kyorin University School of Medicine, 6-20-2 Shinkawa, Mitaka, Tokyo 181-8611, Japan*

[2]*Faculty of Nursing, Tokyo Healthcare University, 2-5-1 Higashigaoka, Meguro, Tokyo 152-8558, Japan*

[3]*Amino Up Chemical Co. Ltd., 363-32 Shin-ei, Kiyota, Sapporo, Hokkaido 004-0839, Japan*

[4]*Department of Health Science, Asahikawa Medical University, 2-1-1-1 Midorigaoka-Higashi, Asahikawa, Hokkaido 078-8510, Japan*

[5]*Social Medical Corporation, the Yamatokai Foundation, 1-13-12 Nangai, Higashiyamato, Tokyo 207-0014, Japan*

E-mail addresses: shirato@ks.kyorin-u.ac.jp (K. Shirato); t-kouda@thcu.ac.jp (T. Koda); takanari@aminoup.co.jp (J. Takanari); junetsu@asahikawa-med.ac.jp (J. Ogasawara); sakutaku@ks.kyorin-u.ac.jp (T. Sakurai); ohnoh2o@yamatokai.or.jp (H. Ohno); kizaki@ks.kyorin-u.ac.jp (T. Kizaki)

Correspondence should be addressed to Ken Shirato; shirato@ks.kyorin-u.ac.jp

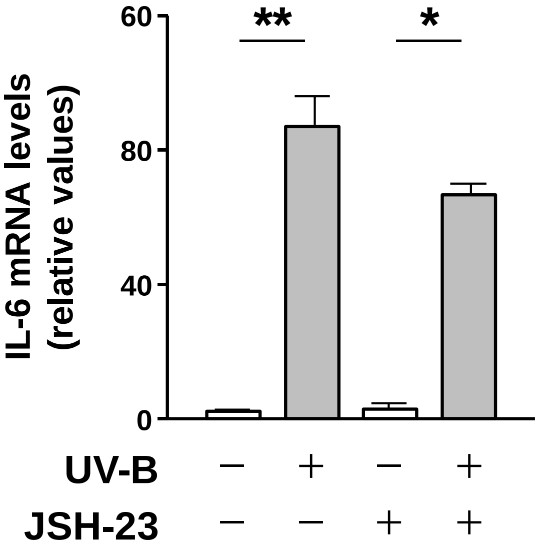

SUPPLEMENTARY FIGURE 1: Effect of an NF-κB inhibitor JSH-23 treatment on UV-B irradiation-induced IL-6 mRNA expression in NHDFs. Cells were treated with 10 μM JSH-23 or dimethylsulfoxide vehicle control for 24 h after UV-B irradiation (20 mJ/cm^2). The mRNA levels of IL-6 were analyzed by real-time PCR. The expression levels of IL-6 mRNA were calculated as the ratio of its value to that of 18S rRNA. Mean ± SEM (n = 3). *$p < 0.05$, **$p < 0.01$ (by one-way ANOVA and the Tukey's test).
